# A case study in participatory science with mutual capacity building between university and tribal researchers to investigate drinking water quality in rural Maine

**DOI:** 10.1016/j.envres.2020.110460

**Published:** 2020-11-17

**Authors:** Tchelet Segev, Abigail P. Harvey, Asha Ajmani, Christopher Johnson, William Longfellow, Kathleen M. Vandiver, Harold Hemond

**Affiliations:** a Civil and Environmental Engineering Department, Massachusetts Institute of Technology (MIT), Cambridge, MA, USA; b Center for Environmental Health Sciences (CEHS) and MIT Superfund Research Program, MIT, Cambridge, MA, USA; c Sipayik Environmental Department, Passamaquoddy Tribal Government, Pleasant Point, Maine, USA

**Keywords:** Citizen science, Water quality, Community engagement

## Abstract

**Background:**

Participatory science or citizen science is increasingly being recognized for providing benefits to scientists and community members alike. However, most participatory science projects include community researchers only in the sample collection phase of the research project. Here we describe how a rural tribal community and urban university utilized participatory science methods to engage community researchers across an entire research study, creating numerous opportunities for mutual capacity building.

**Objectives:**

Researchers from MIT and the Sipayik Environmental Department, a tribal government department, partnered to co-launch a participatory science project to analyze municipal and private well drinking water quality in households in three Maine communities. The objective was to provide households with information about metals, primarily lead and arsenic, in their drinking water, and to improve public education, community partnerships, and local scientific capacity.

**Methods:**

MIT and Sipayik researchers engaged local communities through public community meetings, mailed flyers sent to residents, and meetings with local stakeholders. MIT and community researchers worked together to design and implement the study to quantify metals in community drinking water samples, as well as hold capacity-building trainings. Individual drinking water results were communicated to households, and generalized results were discussed at community meetings in the report-back phase.

**Results:**

The study attained a 29% household participation rate in the region. The researchers completed the analysis and report-back on 652 water samples. Isolated incidences of lead and geologically-attributable arsenic exceeding EPA standards were found. Individual report-backs of the results enabled local participatory scientists to make their own informed public health decisions. The study produced methodologies for navigating potential ethical issues, working with diverse communities, and collaborating over challenging geographical distances.

**Discussion:**

This project developed methodologies to build long-term relationships with local scientists and to engage community members and enhance the environmental literacy of rural communities. Both MIT and Sipayik researchers learned from each other throughout the project; Sipayik researchers built technical capacity while MIT researchers gained local and cultural understanding. Community outreach methods were most effective when sent directly to residents as mailed flyers or through Sipayik researchers’ outreach.

## 1. Introduction

Support for citizen science studies, referred to as participatory science throughout this paper, has risen in recent years due to the benefits community involvement can provide to both scientists and communities. However, these projects often struggle to garner research support and to integrate community members and their interests into the research process (Buytaert et al., 2014; Conrad and Hilchey, 2011; Ramirez-Andreotta et al., 2015; Resnik et al., 2015). Community engagement can have far-reaching benefits, including research acceleration, an increased ability to identify and meet social needs and prevent environmental injustices, and an enhanced public STEM education (Crowdsourcing and Citizen Science Act, 2017; Redmon et al., 2020). Researchers may also benefit from community support when collecting data that spans across a large geographic region and from an enhanced understanding of community needs and interests that can inform the scientific process (Conrad and Hilchey, 2011; Entwistle et al., 1998; Tulloch et al., 2013). Furthermore, increased scientific awareness among non-scientists, termed democratization of science, can engage the community with the environment. Community engagement can promote better environmental management, the inclusion of communities in environmental management decisions, and the ability to influence policymakers (Conrad and Hilchey, 2011). However, while participatory science has important social implications and potential, it also presents numerous challenges.

Participatory science faces challenges regarding setting expectations for research outcomes, uncertainties in the data, and addressing the negative perceptions of community-based research (Buytaert et al., 2014; Conrad and Hilchey, 2011). Additional challenges include ensuring extensive community outreach to the target population, navigating ethical questions (Resnik et al., 2015), and communicating scientifically complex concepts to the general public, concepts such as the potential temporal variation of results (Evans et al., 2005). The utilization of participatory science in water-related research is also limited by the technical complexity associated with the numerous testing and monitoring techniques now available (Buytaert et al., 2016). As a result, participatory science projects lack community participation in the sample analysis phase due to the training necessary to meet the scientific complexity (Ramirez-Andreotta et al., 2015) and the lack of access to the expensive instrumentation or facilities required for the work.

However, there are multiple frameworks to aid the researcher in successfully implementing participatory science projects. The National Institute for Environmental Health Sciences (NIEHS) defines a form of participatory science known as community-based participatory research (CBPR). CBPR aims to incorporate community involvement throughout the research process and intervention methodologies (O’Fallon and Dearry, 2002). Additionally, the Crowdsourcing and Participatory Science Act defines participatory science as a voluntary collaboration between individuals or organizations participating in the scientific process, ranging from formulating research questions and conducting experiments to analyzing data and interpreting results (Crowdsourcing and Citizen Science Act, 2017). For this study, a participatory scientist refers to a member of the public involved in any part of the research, with the form(s) of involvement varying depending on the stage of the project (Table 1).

Another approach is polycentric governance, which involves multiparty responsibilities, decision-making, and generation of information to enhance data trustworthiness and the level of cooperation and participation in participatory science projects (Buytaert et al., 2016). Previous NIEHS research proposed six key principles to a successful CBPR project. These principles include: 1) promoting active collaboration and participation at every stage of research; 2) fostering co-learning; 3) ensuring projects are community-driven; 4) disseminating results in useful terms; 5) ensuring research and intervention strategies are culturally appropriate; and 6) defining community as a unit of identity (O’Fallon and Dearry, 2002). Another framework for approaching participatory science work is Environmental Research Translation (ERT). This five step framework involves creating a trans-disciplinary team to conduct research, ensuring effective collaboration occurs, establishing bidirectional communication through information transfer, incorporating public participation in environmental projects (including co-learning and co-production of data), and developing a cultural model of risk communication for sharing results with the community (Ramirez-Andreotta et al., 2015).

Here, we present a case study of a participatory science project under MIT’s Superfund Research Program (SRP) that implemented the CPBR and ERT frameworks through an original study with unknown contaminant levels in response to community concerns. By incorporating aspects of the CPBR and ERT frameworks, we were able to execute a participatory science project in partnership with underserved communities, overcome sociocultural impediments, co-initiate the project, and build scientific capacity with local tribal organizations and community members. While this research was conducted through an SRP, our study was not focused on one pre-identified contaminated SRP site, but rather as a pilot project for practicing these frameworks in communities while responding to our tribal partner’s environmental health question: “Is our municipal water system making us sick? We have a lot of cancer cases.”

**Fig. 1.**
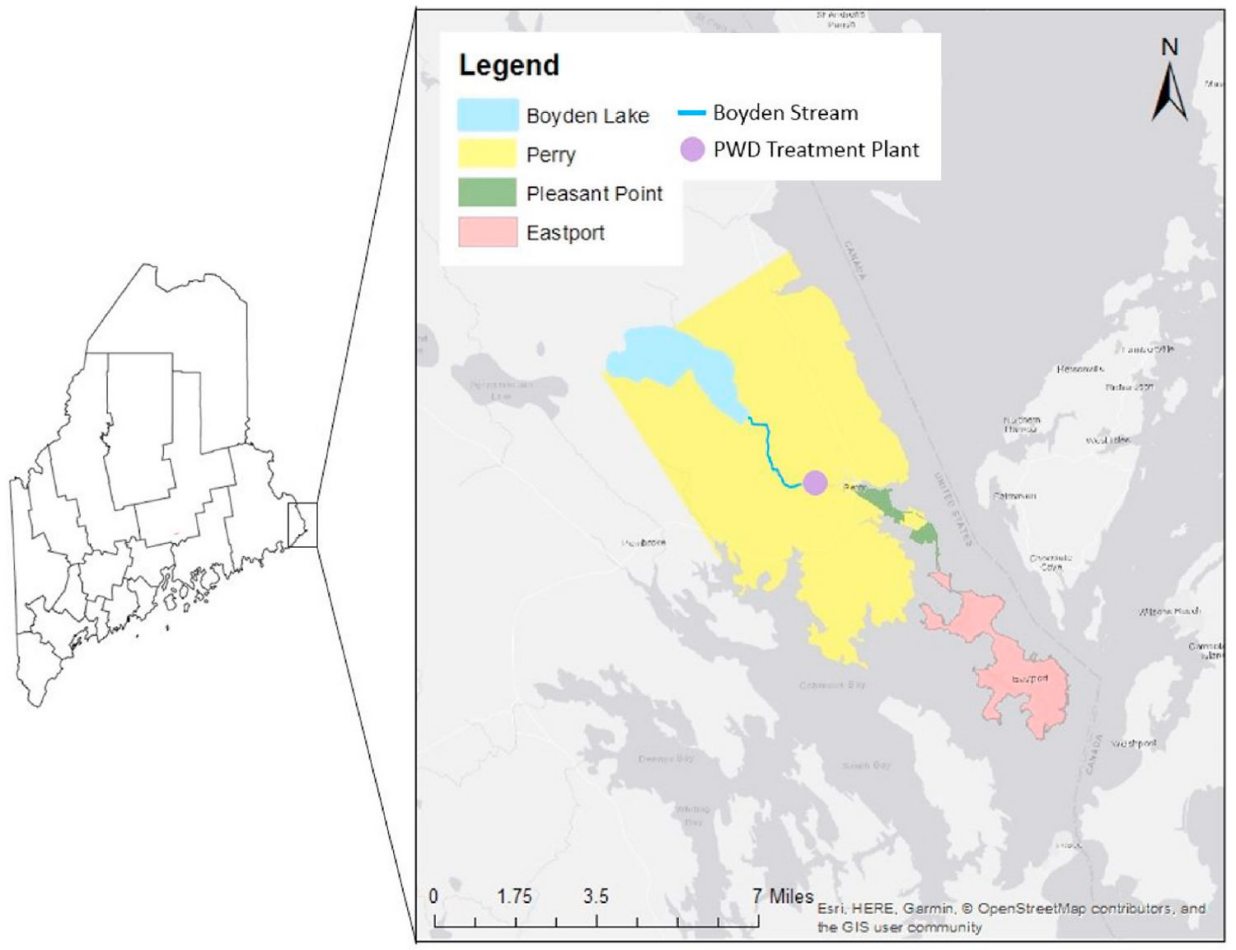
Map of participatory science project area.

This study implemented a participatory science project for three remote coastal communities in northeastern Maine: Eastport, Perry, and Pleasant Point, a village on a Passamaquoddy reservation (Fig. 1). Eastport has a large seasonal population present only in the summer, with a main industry of fishing. Eastport residents rely on a combination of well and/or municipally supplied water, depending on the household. Perry, a rural farming community, relies solely on well water, while Pleasant Point, a Native American reservation of the Passamaquoddy Tribe, relies almost exclusively on the municipal water supply from the Passamaquoddy Water District (PWD) facility. These three communities are diverse socioeconomically, as 45% of the population in Pleasant Point lives below the poverty line compared to 18.8% in Eastport and 19.2% in Perry (US Census Bureau (2000)). The study began in August of 2017 and concluded in June of 2018. The project was designed to solicit input from the community members as participatory scientists, and to engage them in collecting drinking water samples from their own homes. Thus, the drinking water samples in this study would include households with private wells as well as municipally sourced water.

Both lead and arsenic have strict EPA standards for drinking water that may be more often exceeded in communities of lower socioeconomic status (Balazs and Ray, 2014). Studies have found a correlation between lower socioeconomic status and elevated blood lead levels (Kim et al., 2018; Liu et al., 2012; Naicker et al., 2010), and between arsenic intake and socioeconomic status (Argos et al., 2007; Rosado et al., 2007). The water samples submitted from the communities in this study were analyzed for lead, arsenic, and other metals, because they pose health risks that may include neurological defects, cancer, kidney damage, and gastrointestinal distress (Jaishankar et al., 2014). Additionally, previous research indicated the potential for discovering arsenic exceedance of EPA standards in well water samples in this region due to high geogenic arsenic in New England bedrock (Ayotte et al., 2003, 2017). The existence of numerous houses predating the 1986 ban of lead solder in piping also suggested the potential for finding lead-contaminated drinking water in the older homes in the three communities (Nielsen et al. 2010).

This project had two major goals, namely obtaining scientific water quality data and then sharing the water quality data with community members to inform public health decisions. The scientific goal entailed: 1) identifying the concentration of metals at their source, the well or municipal water supply, and at point of use at the household tap; 2) identifying the potential health impacts by conducting a risk analysis assessment; 3) identifying the potential sources of lead and arsenic and 4) providing information on remediation. Through this multi-faceted research, we learned that participatory science can serve as a methodology to address the uncertainty surrounding the quality of publicly or privately owned water sources, and it also can provide a method for successful collaboration with concerned residents to generate information on drinking water quality that both scientists and residents trust.

## 2. Methods

**Fig. 2.**
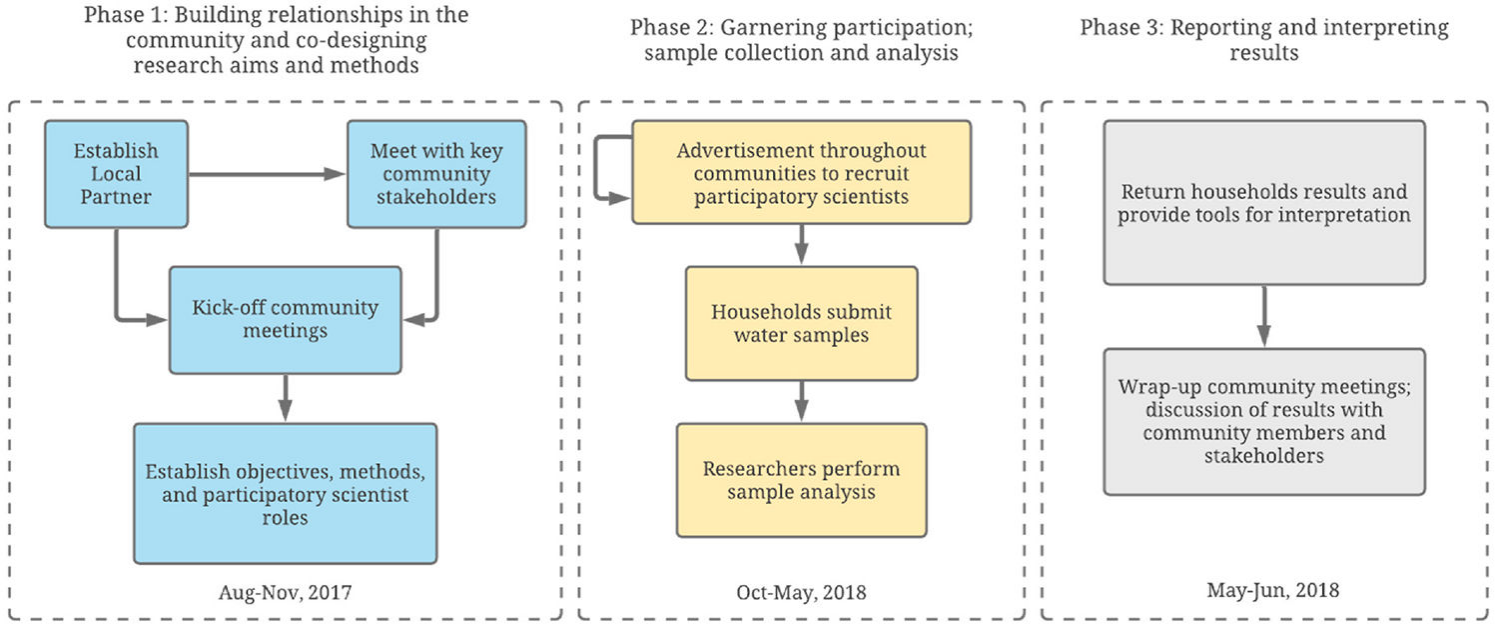
Overview of the participatory science methodology.

Participants in this study are identified in Table 1. There were two types of participants in this study, referred to as “Sipayik researchers” and “participatory scientists,” respectively. The Sipayik researchers’ group includes three co-authors who are members of the Sipayik Environmental Department (SED), a department of the Passamaquoddy Pleasant Point Tribal Government, and who were involved in every stage of the research. The second group, “participatory scientists,” includes members of the three communities who participated in the study, primarily as interested citizens, collecting tap water samples and contributing other essential qualitative data such as type of water supply. A graphic representation of the study methodology is depicted in Fig. 2.

### 2.1. Local tribal partnerships and the research team

Prior to conducting community outreach, MIT and Sipayik researchers held discussions so MIT researchers could gain a better understanding of community concerns about the local environment. Of note, the MIT and Sipayik researchers were already well connected, given that the Community Engagement Core Director for both of MIT’s Center for Environmental Health Sciences (CEHS) and Superfund Research Program (SRP) had already been working with the Passamaquoddy Tribe at Pleasant Point for more than three years. The project discussion was initiated by two graduate students from MIT’s Civil and Environmental Engineering Department who were seeking a thesis topic project that would also benefit an environmental justice community. The numerous and long-standing community concerns about the municipally sourced drinking water rose to the forefront of the conversation. Thus, the Passamaquoddy Tribe’s drinking water research project began to take shape. The water testing was refined to utilize inductively-coupled-plasma mass spectroscopy (ICP-MS) for measuring metals in water.

This research focus matched Sipayik and MIT researcher interests as well as community concerns. The communities in this region have had concerns about their drinking water quality since at least 1986 (Harvey, 2018). Three communities in northeastern Maine were chosen: Eastport, Perry, and Pleasant Point. These communities share the same municipal water source, the Passamaquoddy Water District (PWD), which is not tribally owned; many residences were instead served by private wells. Water flows from Boyden Lake through Boyden stream to the PWD treatment facility, before distribution to houses (Fig. 1). Pleasant Point, a Passamaquoddy reservation, was the focal point for the decision of which communities to include, as the MIT researchers wanted to provide environmental health data that would be useful to a minority environmental justice community. The SED, which serves all tribal lands and waters within Passamaquoddy traditional territory, including Pleasant Point, provided access to this community.

The partnership between MIT and Sipayik researchers was instrumental in this project’s success and included bidirectional capacity building. MIT and Sipayik researchers colaunched the project and had regular two-way communication throughout the project, in person, over the phone, and through email. Three Sipayik researchers, including one non-tribal and two tribal members, were an integral part of the research team, and the goals were discussed and tentatively established, pending community input, with these researchers prior to hosting community meetings. MIT researchers conducted most of the sample analysis, utilizing the ICP-MS to detect metal concentrations, but MIT researchers also trained the Sipayik researchers over three visits on the ICP-MS and sample preparation techniques, including microwave digestion and other lab and safety skills. Hence, the project improved the capacity of the SED to execute their own future research projects while strengthening the SED’s relationship with MIT’s CEHS and SRP. Through handson experience and discussions with the Sipayik researchers, the MIT researchers learned how to engage local participatory scientists, including publicizing the project, communicating complex scientific concepts to the public, and collaborating with local stakeholders and learning about tribal customs.

Individual meetings were also held with other stakeholders in the communities, including local water quality activists, the Passamaquoddy Water District and their engineering consultants, and the local newspaper. The goals were to build trust and establish communication channels, discuss the intent of the study, and highlight that the study was unaffiliated with any agenda except the independent scientific research and community engagement foci. MIT and Sipayik researchers shared updates on the status of the study with these entities regularly to both maintain relationships and educate participatory scientists.

### 2.2. Promoting participation

The study launched with community meetings held in fall and winter 2017. Meetings were held in each participating community to ensure outreach and accessibility to each town, and to include both tribal and non-tribal residents. Meetings were held in August, October, and December 2017 in Eastport, Perry, and Pleasant Point, respectively, in community centers and a school. Following conclusion of the study, two result report-back meetings were held in spring of 2018, in Eastport and Pleasant Point.

During the fall community meetings, the following information was shared: the purpose of the research, the regional area to be included in the study, the study’s focus on metals, the project goals and timeline, the role of participatory scientists, and sample collection procedures. Researchers demonstrated sampling procedures, then distributed sample collection kits. We also solicited input from community members about the chemicals they were concerned about and why, and the responses to our questions were collected first by table groups and then shared back with the entire gathering (Fig. S1). The three questions discussed were: 1) What questions do you have for us? 2) What water quality concerns do you have? 3) What information would you like to share with us?

After the initial community events in each of the three towns, extensive advertisement was used to promote the project. Sipayik researchers put up posters (Fig. S2) at community gathering points such as restaurants, barbershops, and supermarkets, and advertisements were placed in the local newspaper (The Quoddy Tides, 2018). Flyers were also mailed to all US Postal Service addresses in the towns of Eastport, Perry, and Pleasant Point (Fig. S3) at the suggestion of a participatory scientist who used this delivery method to advertise her local business. Our postal flyer outlined the study’s purpose and described the “free” water sampling kits and testing procedures for households and the locations where the kits could be picked up and returned. Outreach on local Facebook groups was also used to keep the community updated on the project (MIT CEHS Boyden Lake Water Quality, 2017). We also relied heavily on word of mouth and in-person outreach through SED researchers to increase participation rates, particularly in the tribal community of Pleasant Point. When initial efforts yielded low participation rates, the deadline for sample submission was extended, and samples from Pleasant Point were accepted on a rolling basis until the completion of the laboratory testing phase.

### 2.3. Identifying Metals of Concern

Metals for the research were selected based on input from Sipayik researchers and community members, as well as from existing data on metal presence in the region, primarily from geological sources. Given documented widespread academic and government public health concerns for this county, arsenic and lead were the primary metals of interest. Well and municipal drinking water samples were also tested for the following metals: copper, manganese, iron, cadmium, zinc, aluminum, selenium, cobalt, nickel, chromium, arsenic, and lead.

### 2.4. Sample collection

Participatory scientists collected the drinking water samples from individual homes, most often their own household. MIT and Sipayik researchers also sampled water and sediment from Boyden Lake and Boyden Stream to test source water quality. We also collected samples from local springs utilized by community members for drinking water. For household sampling, collection kits were distributed to participatory scientists at community meetings and two pickup locations. Each kit contained two 250 mL HDPE bottles for collecting two types of samples. The first sample was a first-draw sample, designated as “standing,” and the instructions were to collect a sample after the tap had not been used for at least 6 h (Fig. S4). The second sample was a flushed sample, designated as “running,” and was collected after the tap had been flushed for 2 min. Participatory scientists filled out labels on the bottle with their name, address, email, phone number, optional GPS coordinates, circled the water source: “Well”, “Passamaquoddy Water District (PWD)”, or “Other” and included date and time of sample collection. Labeling tape colors (red or green) marked the sample as standing or running sample and this designation was confirmed on the label. Participatory scientists could pick up and drop off these sampling bottles in Ziploc bags at two sites, a hardware supply store in Eastport and the Tribal Office in Pleasant Point. Both sites were recommended by Sipayik researchers for their central locations in the respective communities, and the Tribal Office was chosen to promote tribal participation, since it is located on the reservation. Sipayik researchers collected and refrigerated samples within a week of drop-off, and samples were kept refrigerated except during transportation to the MIT lab.

### 2.5. Sample analysis

Samples were analyzed by Inductively Coupled Plasma Mass Spectrometer (ICP-MS). Samples were acidified to 3.5% nitric acid and a Rhodium internal standard was added to all samples to 1 ppb. A stock solution (VHG Laboratories) of aluminum, arsenic, cobalt, chromium, iron, manganese, nickel, lead, zinc, cadmium, and selenium was used for creating the standard curve. Calibration solutions were made by a 10-log dilution series ranging from 100 ppt to 10 ppm. A unique log-linear standard curve was calculated for each run. Any sample with a concentration exceeding 7 ppb for arsenic or 8 ppb for lead was replicated to confirm the sample concentration. The detection limit was determined according to EPA 200.8. While this experiment found the detection limit to be less than 0.1 ppb, we chose to treat this value as the detection limit as it was the lowest point of our calibration curve.

Further scientific analyses beyond the scope of this paper were also conducted; see Harvey (2018) for discussion of sources of arsenic and lead in the region, and Segev (2018) for the human health risk assessment.

### 2.6. Conducting ethical research

To ensure the study was conducted ethically and using scientific best practices for communication with the public, the researchers sought and received guidance from the MIT Internal Review Board (IRB) for determining whether the study qualified as human subjects research under the MIT IRB guidelines (see 45 CFR §46.102(e)(1)). A release of household level water quality results could impact public health, as households could use the results to inform their decisions about their drinking water sources. There might also be health and financial impacts: households that were purchasing bottled water might elect to switch to drinking their tap water, or vice versa. Some households might need to replace old piping, or they might elect to install a filter. Additionally, if data from individual households was shared publicly, property values could be impacted. The IRB consultation determined that this water quality research project was not human subjects research, and so we did not need to submit forms for an MIT IRB exempt status. Although the MIT IRB did not require it, to help participants recognize the limitations and benefits of the study, most sample kits included two copies of a consent form: one to be returned to the research team and one for the participatory scientist to keep.

The Region 1 Environmental Protection Agency (EPA) and Maine Center for Disease Control provided additional guidance on potential legal reporting obligations should metals be detected exceeding EPA standards. We shared levels that exceeded the EPA standard or action level, with a focus on lead, with the Region 1 EPA staff for follow up.

Results were released to all participants in the spring of 2018, as had been communicated earlier to community members. The set timeframe for communicating results ensured community expectations surrounding response time were moderated.

### 2.7. Risk communication: household results and the report-back community meetings

Household level results were only shared with the individual household. Results were sent through the U.S. Postal Service using the address provided by the participatory scientists on the sample label. Returned letters or letters with incorrect or incomplete mailing addresses were instead emailed to the recipients or hand-delivered. The letter contained a disclaimer that the analysis omitted other water quality factors, including organic pollutants and microbial contamination (Fig. S5). The letter also included references to assist participatory scientists in interpretation of the results, potential remedial action, factsheets on arsenic and lead, and other resources. We chose not to provide health interpretations of results, and instead provided references to the websites of the EPA and the State of Maine on drinking water quality. For recipients with additional questions about their results, the letters also contained a telephone number for the State of Maine water quality help desk. This state service is available to answer questions and interpret individuals’ water quality results. They also connect households to financial assistance programs to make home filtration devices more accessible.

Generalized community-level results were shared at two community meetings in June 2018, located in Eastport and Pleasant Point. The report-back meetings were advertised by posters, word of mouth, the project Facebook page, and local community groups’ Facebook pages. The Eastport location was convenient for both Eastport and Perry residents, and the meeting at Pleasant Point was accessible to tribal members, some of whom were Perry residents. At these meetings, the purpose, goals, and methods of the project were reviewed and participation data shared. The EPA standards and potential health effects of arsenic and lead were discussed. In addition, the following information was presented: the number of samples exceeding the EPA standards for arsenic and lead, the impact of filtration at the municipal treatment plant, carcinogenic and non-carcinogenic risks, the sources of the metals, how to reduce exposure to specific metals, and how to interpret the results. To help interpret the results, participants had been reminded to bring their copies. Considerable time at each report-back meeting was given to data interpretation. This community “teach-in” was accomplished by selecting a couple of exemplar reports to illustrate the key takeaways. Results of the regional study were also shared and later reported in the local community newspaper (Coopersmith, 2018a).

## 3. Results

A total of 652 samples were submitted, including both the running and standing drinking water samples submitted, and including 322 well water samples and 276 Passamaquoddy Water District samples. “Other” or unlabeled samples make up the remainder of the count. Total household participation rate was 29%. The demographic distribution was 367 from Eastport, 251 from Perry, and 34 from Pleasant Point, corresponding to participation rates of 27% from Eastport, 35% from Perry, and 6% from Pleasant Point. The total tribal participation was adjusted to 15% (up from 6%) when tribal members living outside of the reservation were accounted for in the study. The participation rate among residents unaffiliated with the Passamaquoddy tribe was 34%. This demographic information was calculated using data from the US Census Bureau (2000). Attendance in the first community meeting in Eastport attracted around 40 attendees, while the meetings in Perry and Pleasant Point, the first a community of both tribal and non-tribal members and the latter a town on the reservation, had fewer than 5 attendees each.

First, here is an explanation of the terms and abbreviations used in reference to the EPA standards that will be presented in this section and in Table 2. The EPA standards list the Primary Maximum Contaminant Level (MCL) and Maximum Contaminant Level Goal (MCLG), and for metals such as zinc that had neither values, our study used the Secondary MCL. The Primary MCL is an enforceable standard and is the highest contaminant level allowed in municipal drinking water. The Primary MCLs are set as close to the MCLGs as possible when considering cost and available treatment technology. MCLGs are concentrations of contaminants in drinking water below which there are no known or expected health risks. They include a margin of safety and are non-enforceable targets. As in the case of lead and copper, MCLs can be limited by available Treatment Techniques (TT), which are the required processes to reduce contaminant levels in municipal drinking water systems (Environmental Protection Agency, 2015b). For lead and copper, there are no MCLs, but instead municipal water sources are required to maintain levels below the TT, and must act to reduce levels if they exceed the TT. Secondary MCLs are also non-enforceable standards for municipal water systems to utilize for aesthetic considerations, including taste, odor, and color (Environmental Protection Agency, 2015a).

In the towns of Perry and Eastport, 15% of Perry wells and 10% of Eastport wells exceeded arsenic EPA standards. Additionally, 5 Eastport well samples, 4 Perry well samples, and 2 Eastport municipal water samples had instances of lead exceeding the EPA standards. However, the lead values in running samples for these households dropped, demonstrating the efficacy of running the tap for 2 min in order to reduce lead exposures. Other metals of lesser (but non-zero) potential health impacts, including manganese, aluminum, iron, copper, and zinc, also exceeded EPA standards in isolated cases (Table S55). Table 3 shows the mean, standard deviation, and median for arsenic and lead in different populations and sample types within the study area (Harvey, 2018; Segev, 2018).

## 4. Discussion

Through this study, we aimed to obtain scientific water quality data and share that data with community members to inform public health decisions. Of the three-town area, 29% of households participated in the study. We found that 15% and 10% of wells exceeded EPA guidelines for arsenic in Perry, and Eastport, respectively, and isolated households in Eastport and Perry exceeded EPA guidelines for lead.

We were able to garner these levels of public participation mainly through building relationships and maintaining bi-directional communication with local scientists, activists, and by holding multiple public meetings in all three towns. The relationship with local researchers was instrumental in the success of the study. The most effective action to increase participation was the informational flyer sent to all homes in Eastport, Perry, and Pleasant Point. Participation rapidly spiked following the delivery of flyers through the US Postal Service.

### 4.1. Comparison to peer-reviewed literature

Fifteen percent of wells tested in Perry exceeded EPA guidelines for arsenic, compared to 20% in a previous U.S. Geological Survey (USGS) study in Perry. Given a sample size of 83 households in this study compared to 74 for the USGS, together with testing at different times in a different subset of wells, the difference is not considered significant (Nielsen et al., 2010). Our results also indicated that levels of arsenic and lead exceeding EPA standards were localized to the household level and did not indicate a systemic issue in the water system or concentrated regions of arsenic (Harvey, 2018).

Similar to previous studies, we found that face-to-face meetings such as our community meetings and direct outreach such as the mailed flyers were most effective in increasing participation (Brown et al., 2012; Evans et al., 2005; Ramirez-Andreotta et al., 2015). We also found that committing to a report-back, i.e. informing residents that we will provide study participants with their drinking water results, greatly increased participation, similar to Ramirez-Andreotta et al. (2015). Our study resulted in much greater participation than other community-based participatory research projects, as in the Ramirez-Andreotta study, where only 25 people fully completed the project, and in Brown et al. (2012), where fewer than 50 people participated in the study, compared to over 300 households in our study.

One potential reason for our increased participation may have been our mailed flyers, distributed by the U.S. Postal Service to every household in the three-town area, to advertise the “free” water testing project. Participation more than doubled following receipt of these flyers. The mailed flyers were particularly effective in this area because they were low-cost for the MIT team to produce and reached the majority of the communities’ residents. MIT researchers prepared and printed the flyers, then shipped them to the Sipayik researchers in Maine. The Sipayik researchers then delivered the flyers to local post offices, which delivered them to all registered households in the area. In the flyers, we highlighted the fact that participation in the study was free to participants because water testing may not be something households may otherwise be able to afford. By emphasizing the “free” water quality testing, we were able to greatly increase participation.

Additionally, the time commitment required for participatory scientists in our study was much lower than in Ramirez-Andreotta et al. (2015), as participants in our study needed only to collect samples on one day, drop them off at one of several central locations, and receive their results in the mail and could choose to attend two informational meetings, rather than continued participation spanning months or years as in Ramirez-Andreotta. While this shortened time frame for participation may have reduced the quality of the relationship we formed with our community members, we created long-term academic partnerships by including members of the Sipayik Environmental Department in the entire process of the study and by providing them with training opportunities in our laboratories at MIT.

While we did find some strengths of our project in common with other studies, unique to our project was the formation of long-term partnerships with a local research team. The Sipayik researchers were vital in building MIT researchers’ capacity to engage community members, in particular tribal members, and for identifying local contacts and sample pick-up and drop-off locations, and contributing knowledge of the local culture. Additionally, Sipayik researchers made the collection of 650+ samples possible by committing to pick samples up on-site and deliver them to MIT researchers for analysis; without this contribution, sample collection would have been heavily limited due to transportation logistics.

While crowdsourcing may be common in ecological sciences, studies in hydrology and drinking water rarely incorporate community scientists due to the complexity of hydrological sampling. Another university’s SRP Community Engagement Core project involved collection and analysis of environmental waters through a partnership with the Yurok Tribal Environmental Program. However, engagement with the overall community was limited to only an informational flyer containing results handed out at a prominent tribal event. Community members were not directly involved in collecting samples or determining sampling locations, and thus the participatory science conclusions that can be drawn from this study are limited (Middleton et al., 2019). However, Redmon et al. (2020), recently demonstrated the efficacy of community science in collection of first-draw (non-flushed standing) water samples at child centers and daycares in North Carolina to determine lead concentrations in drinking water (Redmon et al., 2020). More case studies implementing CBPR in water quality studies are needed to further highlight areas of improvement in this field.

### 4.2. Limitations

Overall, we were able to garner wide public participation in our study and disseminate water quality results to all participants through mailed flyers, results letters, and public meetings. However, we faced several challenges due to the large physical distance between MIT researchers and the towns of study and to cultural differences between MIT researchers and tribal members. The most effective methods for engaging the public despite this distance were through the partnership with Sipayik researchers, the mailed flyers, and meetings with various stakeholders.

However, the efficacy of the public meetings in garnering public participation is less clear in this study. While the initial meetings held while the project was still in the planning stage were able to clarify community concerns and garner vital community input into the study aims, attendance was much lower than the total number of study participants (approximately 50 attendees at the initial three public meetings versus >300 total participants). Because participation more than doubled following the mailed flyer, using mailed flyers to advertise public meetings could have also increased attendance at both sets of meetings. Additionally, attendance at the tribal public meetings was very low, and tribal participation was lower than non-tribal participation overall; 15% of tribal members submitted samples, compared to 34% for non-tribal members, despite the heavy involvement of Sipayik researchers in the project. One possible reason for lower tribal attendance at public meetings may be due to MIT researchers serving as the main presenters of the meetings. Future studies may benefit from empowering tribal scientists, such as the Sipayik researchers, to directly lead meetings themselves.

Additionally, while the study provided extensive data on drinking water quality, the results were not exhaustive, as they omitted all non-metals, including chlorination by-products (a community concern; see Fig. S1). Communicating that this water study was not all-inclusive and only analyzed the water for metals and not for other health hazards such as microbial was imperative to the community’s understanding of the reported water quality both on the household and community level. This is exemplified through the local newspaper publishing a story about the results meeting under the title “PWD water deemed safe, according to independent study,” despite the limited range of water quality testing conducted in this study (Coopersmith, 2018b).

## 5. Conclusion

Our results contribute to a growing literature on the benefits of community outreach, and provides a case study of a successful community outreach study. The success of the study is attributable not only to the partnership with the SED, but also to the numerous partnerships formed with other community organizations, including the PWD. We found that establishing partnerships, maintaining communication throughout the project, and inviting the community and individual stakeholders to community meetings were vital to the project’s success.

In combination with the local partnerships, extensive advertising was important to increase participation rates. This was accomplished through community meetings, newspaper advertisements, flyers, and word of mouth. Despite this, acknowledging the need to conduct additional outreach to the tribal community was important to make the opportunity equally accessible to all members of the population. Last, allowing participatory scientists regular and easy access to the researchers, through social media, email, telephone, community meetings, and the Sipayik researchers, increased community and stakeholder trust in the process.

The project relied heavily on communication with individual community members, key stakeholders, and a champion local partner. The success of the project generated a trove of materials and lessons learned that can be capitalized on for the success of future non-traditional participatory science projects.

The success of this participatory science project was perhaps best encapsulated when the participants of this study expressed interest in a continuing the partnership, measuring other chemicals, including disinfection by-products in the drinking water, and also when another tribe in Maine expressed interest in replicating the study in their community (Fig. S1).

## Supplementary Material

1

## Figures and Tables

**Table 1:** The role of participatory science in the scientific process, and associated challenges.

Step in Scientific Process	Participatory Science Role	Limitations
Observe community issues	Community members expressed concern about their drinking water quality	Relied on previously established relationship between MIT and Sipayik researchers through MIT’s CEHS Community Engagement Director meeting with the SED director for several years and through the EPA Region 1 Regional Tribal Operations Committee’s monthly phone calls
Identify study purpose and questions	Sipayik and MIT researchers collaborated to identify study purpose and questions Participatory scientists provided feedback during kick-off community meetings	The scope of the study had to be limited given the researchers’ limited resources and time availability
Research existing information	Participatory scientists contributed anecdotal evidence and reports at community meetings, when discussing question #3, what information would you like to share with us? (Fig. S1)	Availability of relevant online information was limited Anecdotal evidence provided by participatory scientists was difficult to triangulate within the resources of this study Anecdotal evidence was limited to those who self-selected to participate in the study
Develop hypotheses	Discussed in community meetings under question #2, what water quality concerns do you have? (Fig. S1)	Few participatory scientists had conducted research into the local drinking water quality or had tested their drinking water, so hypotheses relied on anecdotal evidence Concerns that PWD stakeholders may be alienated during discussion as participatory scientists had many municipal water quality concerns
Collect samples	Participatory scientists collected and labeled their own household level samples, using the kits provided Sipayik and MIT researchers collaborated to collect the surface water samples	Not possible to verify whether participatory scientists properly followed sample collection instructions for household samples
Analyze samples	Sipayik and MIT researchers collaborated to analyze samples	The technical expertise required for sample analysis limited the participation of all other participatory scientists
Analyze data	Sipayik researchers participated in sample logging	The technical expertise required excluded the involvement of participatory scientists
Interpret data and conduct research translation	Participatory scientists received letters with their results, and references to aid in independently interpreting results Aggregate results were shared at community meetings and discussed by participatory scientists in attendance Results were shared in the local newspaper	The researchers could not provide health related interpretation as it was outside their expertise, despite many participatory scientists’ inquiries. Participatory scientists were encouraged to utilize the references provided Some participatory scientists found it challenging to independently interpret their results with the references Determining contamination sources and public health risk

**Table 2:** EPA MCL and MCLG concentrations for metals of interest (Environmental Protection Agency, 2015a, 2015b).

Metal	Concentration (ppb)		
	Primary MCLG	Primary MCL	Secondary MCL
Lead	0	TT: 15	–
Arsenic	0	10	–
Copper	1300	TT: 1300	1000
Aluminum	–	–	50–200
Chromium (total)	100	100	–
Manganese	–	–	50
Iron	–	–	300
Cobalt	–	–	–
Nickel	–	–	–
Zinc	–	–	5000
Selenium	50	50	–
Cadmium	5	5	–

**Table 3:** Summary statistics for lead and arsenic levels in drinking waters sources for three communities in Maine (Eastport, Perry and Pleasant Point).

Source	N	Arsenic (ppb)				Lead (ppb)			
		min	median	p95	max	min	median	p95	max
Wells									
Overall	141	<0.1	1.4	17.1	40.7	<0.1	0.4	9.4	86.7
Eastport	54	<0.1	1.5	14.7	40.7	<0.1	0.4	7.6	9.8
Perry	86	<0.1	1.4	18.7	34.5	<0.1	0.5	10	86.7
Tribal	12	<0.1	3.3	11.3	17.1	<0.1	0.2	3.2	5.6
Non-tribal	129	<0.1	1.4	18.4	40.7	<0.1	0.5	9.5	86.7
Municipal water (PWD)									
Overall	122	<0.1	0.2	0.6	1.6	<0.1	0.1	4	16.9
Eastport	98	<0.1	0.2	0.6	1.6	<0.1	0.2	4	16.9
Perry	6	<0.1	0.1	0.2	0.3	<0.1	0.2	3.2	3.6
Tribal	24	<0.1	0.1	0.4	0.9	<0.1	<0.1	3.4	4.5
Non-tribal	98	<0.1	0.2	0.6	1.6	<0.1	0.2	4	16.9
