# Supplementary material for: A case study in participatory science with mutual capacity building between university and tribal researchers to investigate drinking water quality in rural Maine
Source: Environ Res. Author manuscript; Available in PMC 2021 Jan 6. (PMC7787195; doi:10.1016/j.envres.2020.110460)
Supplement: 1 [file NIHMS1652100-supplement-1.docx]

**Supporting Information**

**A case study in participatory science with mutual capacity building between university and tribal researchers to investigate drinking water quality in rural Maine**

Tchelet Segev,^*12^ Abigail Harvey,^*12^ Asha Ajmani,^3^ Christopher Johnson,^3^ William Longfellow,^3^ Kathleen M. Vandiver,^2^ Harold Hemond^12^

^*^These authors contributed equally to this work

^1^Civil and Environmental Engineering Department, Massachusetts Institute of Technology (MIT), Cambridge, Massachusetts, USA

^2^Center for Environmental Health Sciences (CEHS) and MIT Superfund Research Program, MIT, Cambridge, Massachusetts, USA

^3^Sipayik Environmental Department, Passamaquoddy Tribal Government, Pleasant Point, Maine, USA

Corresponding author: Abigail Harvey, 77 Massachusetts Avenue, Cambridge, MA 02139

Present address: 200 College Ave, Medford, MA 02155

**Table S1:** Summary statistics for all metals tested in this study: arsenic, lead, cobalt, manganese, iron, cadmium, zinc, aluminum, selenium, cobalt, nickel, and chromium. For results by town or water source, see Segev, 2018 or Harvey, 2018.

| Metal | min | 5^th^ percentile | median | mean | 95^th^ percentile | max |
| --- | --- | --- | --- | --- | --- | --- |
| Arsenic | <0.1 | 0.1 | 0.4 | 2.6 | 14.4 | 40.1 |
| Lead | <0.1 | <0.1 | 0.3 | 1.7 | 5.3 | 86.7 |
| Cobalt | <0.1 | <0.1 | <0.1 | <0.1 | 0.1 | 6.6 |
| Manganese | <0.1 | 0.1 | 0.6 | 43.2 | 45.4 | 4800 |
| Iron | 0.1 | 0.4 | 1.7 | 40.9 | 25.8 | 5700 |
| Cadmium | <0.1 | <0.1 | <0.1 | <0.1 | 0.1 | 0.6 |
| Zinc | 0.2 | 0.5 | 12.8 | 94.4 | 400 | 2600 |
| Aluminum | 0.1 | 0.3 | 4.4 | 17 | 65 | 260 |
| Selenium | 0.1 | 0.1 | 0.2 | 0.3 | 0.5 | 3.5 |
| Cobalt | <0.1 | <0.1 | <0.1 | <0.1 | 0.2 | 6.6 |
| Nickel | <0.1 | <0.1 | 0.6 | 5 | 8.2 | 730 |
| Chromium | <0.1 | <0.1 | 0.1 | 0.1 | 0.3 | 3.9 |

Figure S1: Discussion questions and topics from the Eastport community meeting in August


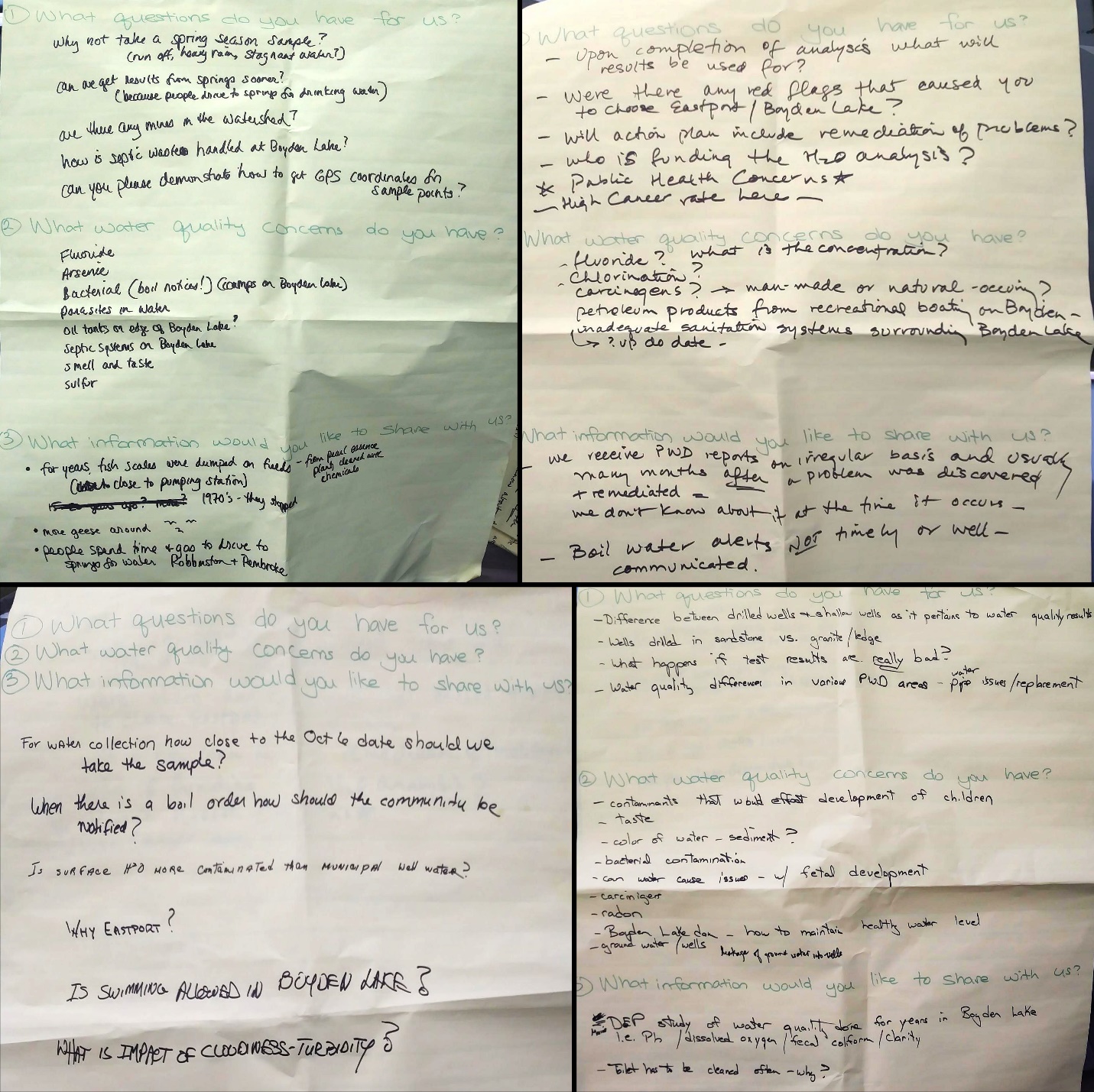


**Figure S2:** Sample posted advertising the October community meeting in Perry, ME


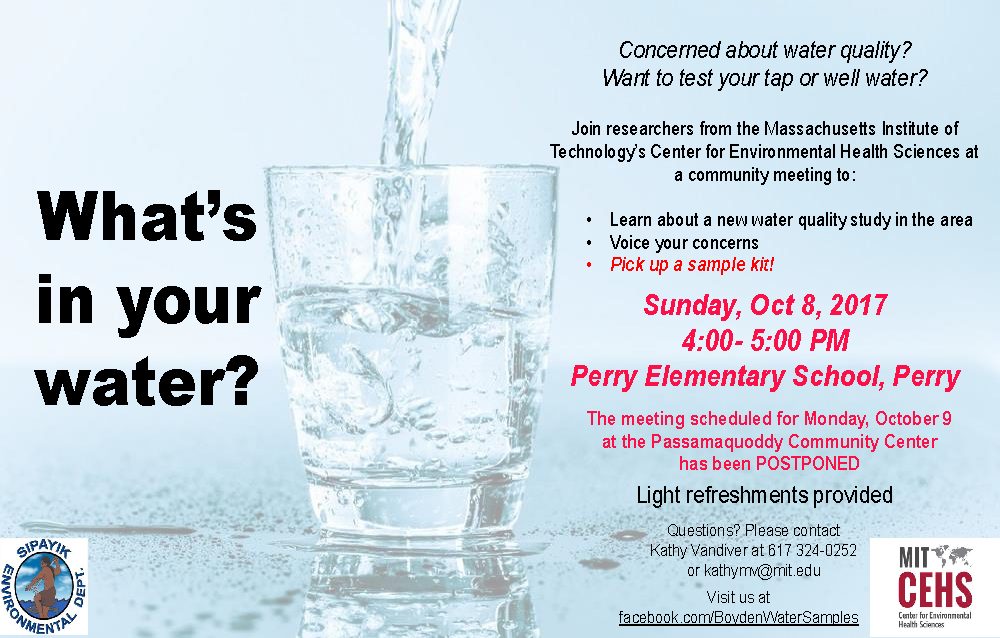


**Figure S3:** Flyer sent to all residents of Perry, Eastport, and Pleasant Point that had a mailing address


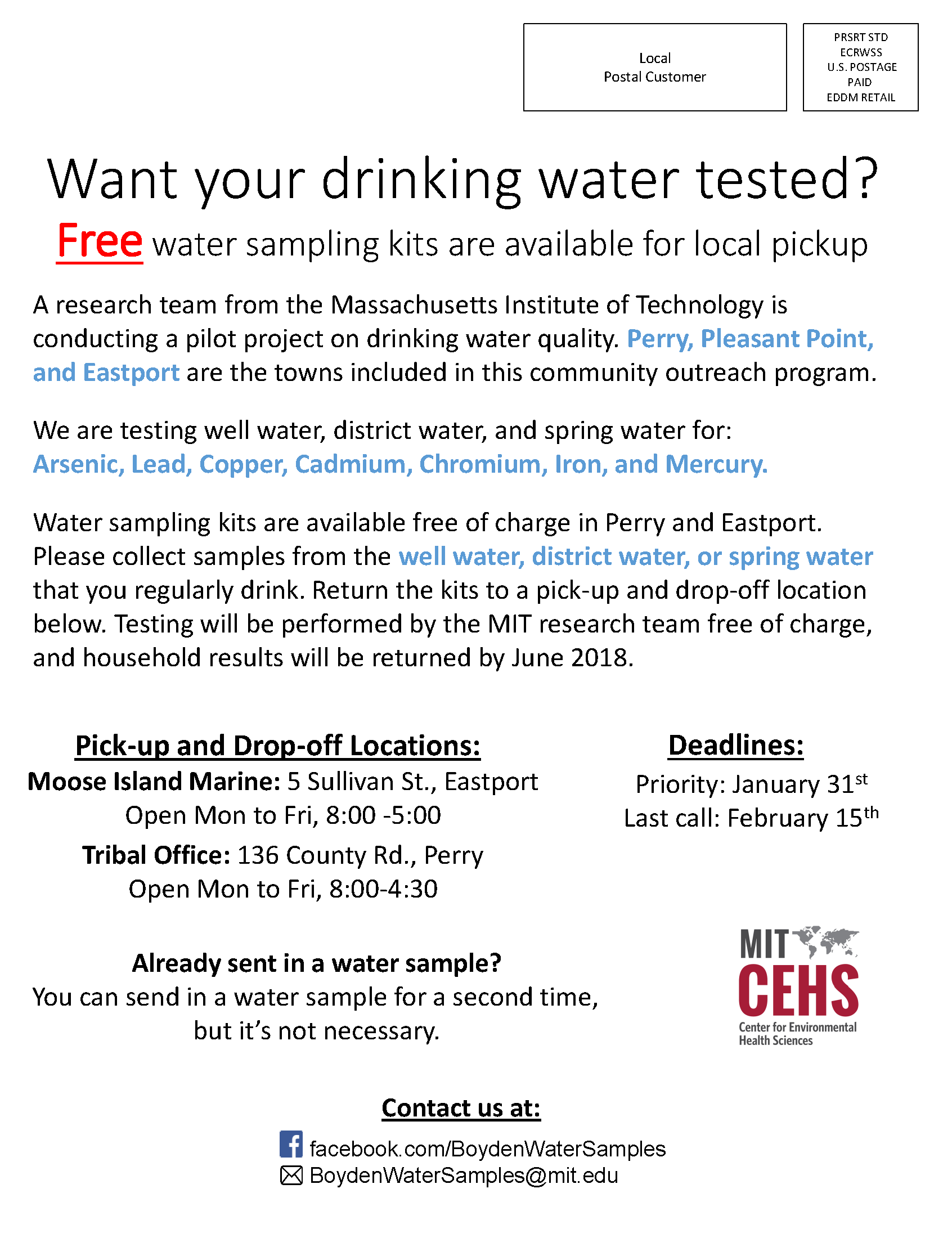


**Figure S4:** Instructions for sample collection included in all kits, and also included on community meeting handouts


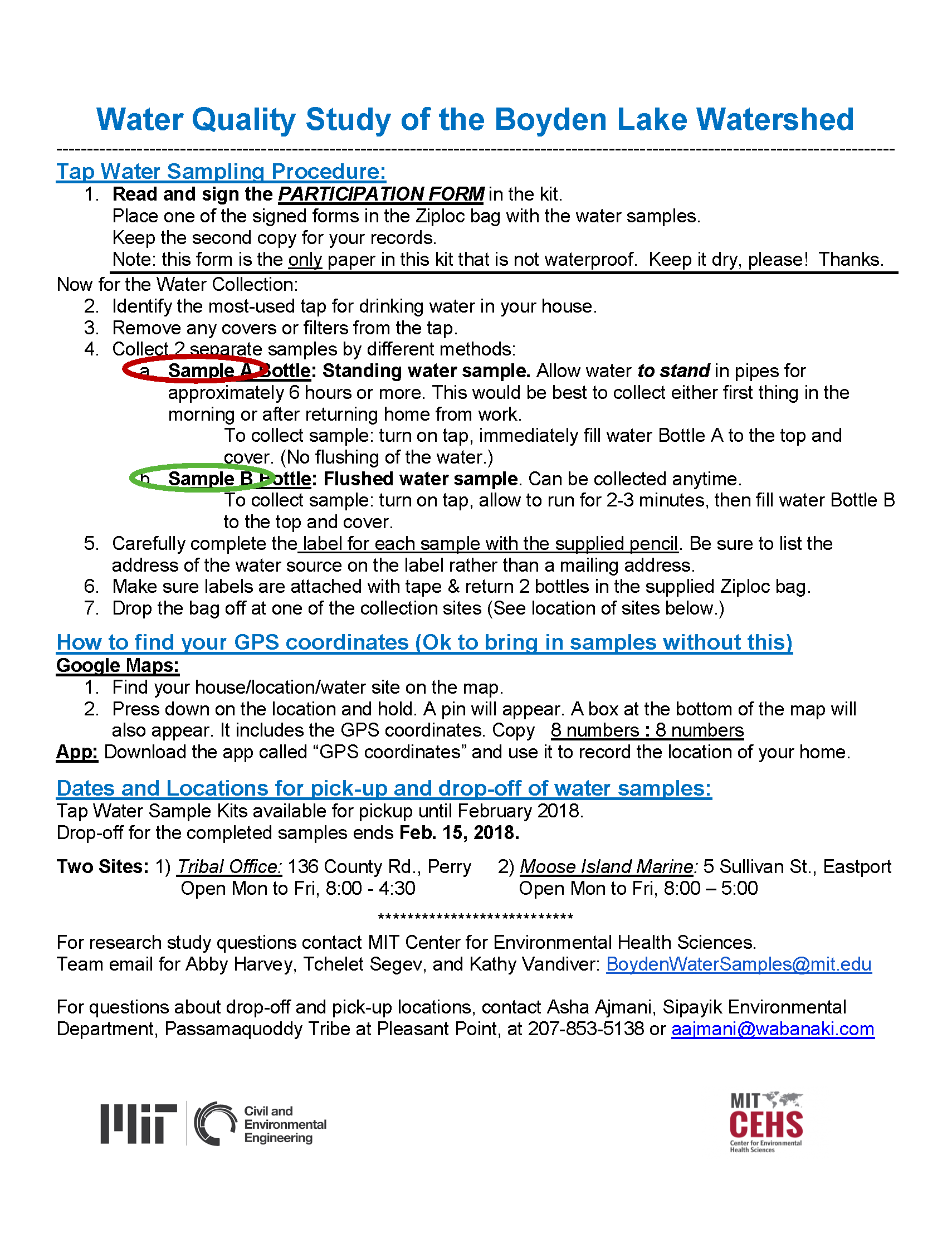


**Figure S5**: Letter template used to return results to participatory scientists

May 23, 2018

Dear «Name_left»,

Thank you for participating in the Water Quality Study of the Boyden Lake Watershed conducted at the Massachusetts Institute of Technology (MIT). We are sending a second copy of results by email for your reference.

On page 3 of this letter, we report the concentrations of metals measured in the «WaterSource_left» water sample you submitted for «Address_left», «AddressTown_left», «AddressState_left» in a data table. The same table also includes the national water quality standards. To help you interpret these numbers, we have provided a list of helpful resources on page 2.

This study was conducted by two Master’s student researchers at MIT’s Civil and Environmental Engineering Department. The study analyzed only specific metals, and did not test for bacteria, organic chemicals, or chlorination byproducts in the water. Therefore, these results provide a partial picture of your drinking water quality.

Thank you again for participating in the study.

Regards,

Abby Harvey and Tchelet Segev

MIT Environmental Engineering Master’s Students

Email: BoydenWaterSamples@mit.edu

Facebook: <https://www.facebook.com/BoydenWaterSamples/>

**Resources**

Below are some websites that provide additional information:

Results Interpretation

For more information on how to interpret your results, health effects, and possible remedial actions, please visit the Ohio Watersheds Network:

<https://ohiowatersheds.osu.edu/know-your-well-water/well-water-interpretation-tool>

Wells

If you own a well, you can call 866-292-3474 (toll-free in Maine) or 207-287- 4311 to talk to an expert about your results and visit wellwater.maine.gov to learn more about well water testing and drinking water quality.

For water with elevated Arsenic levels, you can also refer to the following Arsenic factsheet from the Maine Department of Health and Human Services: <http://www.maine.gov/dhhs/mecdc/environmental-health/eohp/wells/documents/arsenicresultstipsheet.pdf>

For more information about arsenic in well water, you can refer to arsenicandyou.org.

Maine Housing’s Arsenic Abatement Program provides grants to eligible single-family homeowners or landlords with private well water if there is evidence of high levels of arsenic contamination. You can find more information about the program here: <http://mainehousing.org/programs-services/HomeImprovement/homeimprovementdetail/arsenic-abatement-program>

Lead in Water

For water with elevated Lead levels, please refer to the following factsheet from the Environmental Protection Agency: <https://www.epa.gov/ground-water-and-drinking-water/basic-information-about-lead-drinking-water>

Email: BoydenWaterSamples@mit.edu

Facebook: <https://www.facebook.com/BoydenWaterSamples/>

**Results**

Below are the results for your standing and flushed samples.

- The standing sample is water that stood in pipes for at least 6 hours, and shows the effects of piping water quality.
- The flushed water sample is the water you collected after letting the tap run for 2-3 minutes, and shows your water quality without the effect of the piping.
- The Primary Standard Maximum Contaminant Level is the highest level of a contaminant allowed in drinking water to protect the public health.
- Secondary Standard Maximum Contaminant Levels are optional water quality standards established for considerations such as taste, color, and odor. These contaminants do not present a risk to human health.
- The results are reported in units of micrograms per liter (µg/L).

|  | **Your Results** | | **EPA Standards** | |
| --- | --- | --- | --- | --- |
| Name of Metal | Standing Sample Concentration (µg/L) | Flushed Sample Concentration (µg/L) | EPA Primary Standard Maximum Contaminant Level (µg/L) | EPA Secondary Standard Maximum Contaminant Level (µg/L) |
| Lead (Pb) |  |  | **15** | **None** |
| Arsenic (As) |  |  | **10** | **None** |
| Copper (Cu) |  |  | **1300** | **1000** |
| Manganese (Mn) |  |  | **None** | **50** |
| Iron (Fe) |  |  | **None** | **300** |
| Cadmium (Cd) |  |  | **5** | **None** |
| Zinc (Zn) |  |  | **None** | **5000** |
| Aluminum (Al) |  |  | **None** | **50-200** |
| Selenium (Se) |  |  | **50** | **None** |
| Cobalt (Co) |  |  | **None** | **None** |
| Nickel (Ni) |  |  | **None** | **None** |
